# 248. QuantiFERON TB Gold Test Utilization during Inpatient and Emergency Department Encounters at a Multi-Site, Single Academic Institution

**DOI:** 10.1093/ofid/ofaf695.091

**Published:** 2026-01-11

**Authors:** Laxman Singanamala, Devin Weber, Laura Walters, Courtney E Comar

**Affiliations:** Thomas Jefferson University, Philadelphia, Pennsylvania; Department of Medicine, Division of Infectious Diseases, Sidney Kimmel Medical College at Thomas Jefferson University, Philadelphia, PA; Thomas Jefferson University Hospital, Glenside, Pennsylvania; Thomas Jefferson University, Philadelphia, Pennsylvania

## Abstract

**Background:**

The QuantiFERON-TB Gold Plus test (QG) is an in vitro Interferon Gamma Release Assay used to assess prior exposure to Mycobacterium tuberculosis (MTB), primarily for latent TB screening. TB remains a major global health concern, with an estimated one-quarter of the world's population infected and a global incidence of 127 cases per 100,000 (WHO). In contrast, the U.S. has a low incidence—2.5 cases per 100,000 in 2022 (CDC). Despite this, QG was frequently used at our institution in Philadelphia, PA. We sought to understand our patient population and clinical rationale for testing, aiming to reduce unnecessary testing and associated costs by optimizing the QG order set in the electronic medical record (EMR).

**Table 1.**
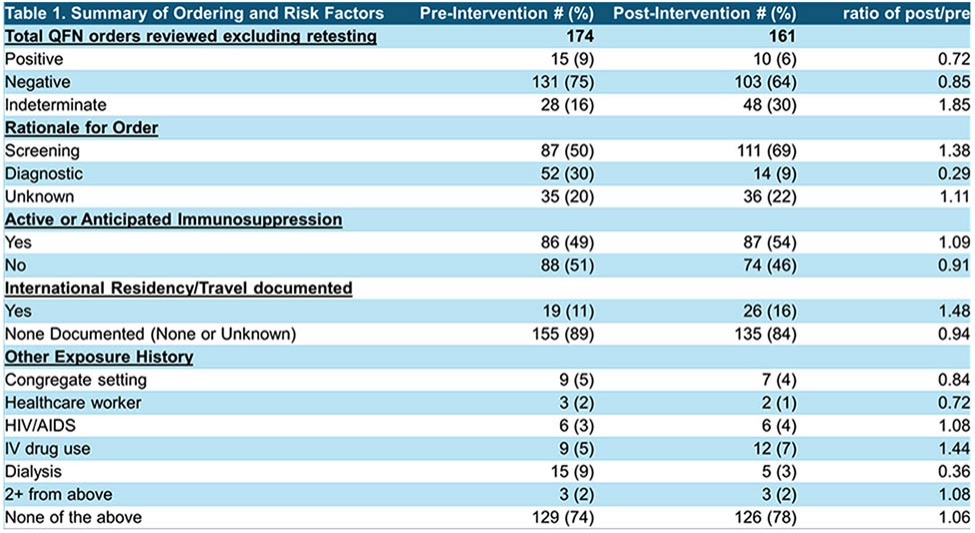
Summary of Ordering and Risk Factors

**Figure 1.**
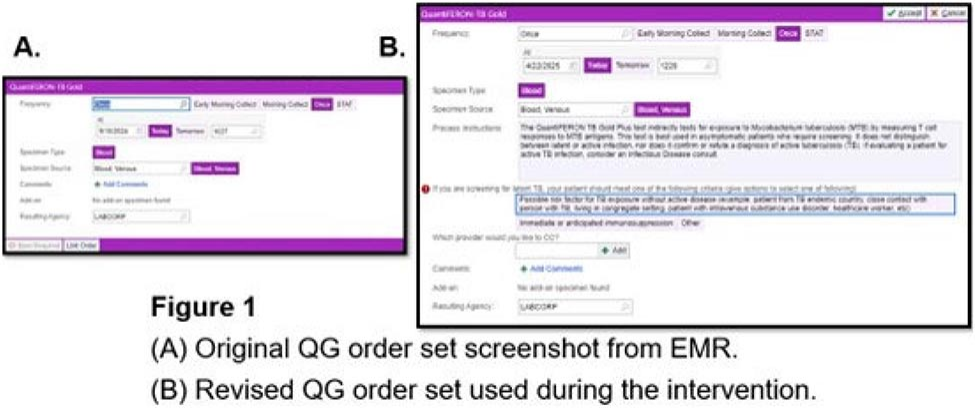
(A) Original QG order set screenshot from EMR (B) Revised QG order set used during the intervention

**Methods:**

Using the Plan-Do-Study-Act (PDSA) framework, we reviewed 2 months of QG orders from inpatient and emergency visits across several hospitals in Pennsylvania and New Jersey before implementing changes. Over 30% of orders were inappropriate based on author review using guidelines from the Infectious Diseases Society of America and the U.S. Preventive Services Task Force. As an intervention, we modified the EMR QG order to include radio buttons guiding clinicians toward appropriate indications (Figure 1). We then reviewed QG orders from 2 months post-intervention.

**Figure 2.**
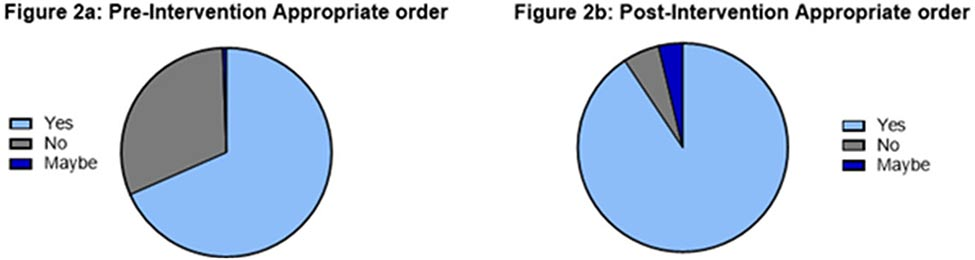
2a: Pre-intervention appropriate order 2b: Post-intervention appropriate order

**Figure 3.**
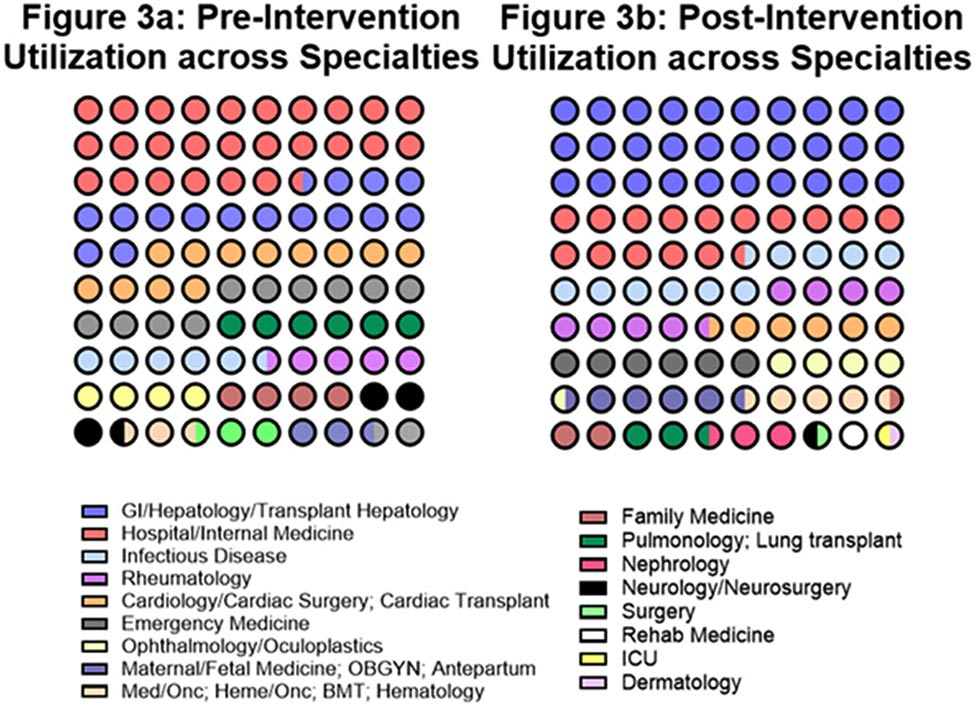
3a: Pre-intervention utilization across specialties 3b: Post-intervention utilization across specialties

**Results:**

While overall QG ordering volume remained stable (Table 1), the proportion of clinically inappropriate orders declined significantly (Figure 2b). A large portion of patients required QG testing for planned or active immunosuppression (49% pre-intervention, 54% post-intervention). Incidence of latent/active TB and patient outcomes were recorded but lacked power for significance.

**Conclusion:**

After implementing EMR changes, inappropriate QG testing declined. We gained insight into our patient population and found QG was often embedded in non-specific order sets. Poor documentation of risk factors may have influenced chart review accuracy. Importantly, this project shows how Infectious Disease and Microbiology teams can collaborate to promote diagnostic stewardship—reducing unnecessary tests, improving patient care, and lowering costs.

**Disclosures:**

All Authors: No reported disclosures

